# Screening of Chorioamnionitis Using Volatile Organic Compound Detection in Exhaled Breath: A Pre-clinical Proof of Concept Study

**DOI:** 10.3389/fped.2021.617906

**Published:** 2021-05-26

**Authors:** Daan R. M. G. Ophelders, Agnes W. Boots, Matthias C. Hütten, Salwan Al-Nasiry, Reint K. Jellema, Owen B. Spiller, Frederik-Jan van Schooten, Agnieszka Smolinska, Tim G. A. M. Wolfs

**Affiliations:** ^1^Department of Pediatrics, Maastricht University Medical Center+, Maastricht, Netherlands; ^2^GROW School for Oncology and Developmental Biology, Maastricht University, Maastricht, Netherlands; ^3^Department Pharmacology and Toxicology, Maastricht University, Maastricht, Netherlands; ^4^NUTRIM School of Nutrition and Translational Research in Metabolism, Maastricht University, Maastricht, Netherlands; ^5^Department of Obstetrics and Gynecology, Maastricht University Medical Center+, Maastricht, Netherlands; ^6^Division of Infection and Immunity, School of Medicine, Cardiff University, Cardiff, United Kingdom

**Keywords:** preterm birth, non-invasive diagnostics, exhaled breath volatile organic compound, chorioamnionitis, biomarker

## Abstract

Chorioamnionitis is a major risk factor for preterm birth and an independent risk factor for postnatal morbidity for which currently successful therapies are lacking. Emerging evidence indicates that the timing and duration of intra-amniotic infections are crucial determinants for the stage of developmental injury at birth. Insight into the dynamical changes of organ injury after the onset of chorioamnionitis revealed novel therapeutic windows of opportunity. Importantly, successful development and implementation of therapies in clinical care is currently impeded by a lack of diagnostic tools for early (prenatal) detection and surveillance of intra-amniotic infections. In the current study we questioned whether an intra-amniotic infection could be accurately diagnosed by a specific volatile organic compound (VOC) profile in exhaled breath of pregnant sheep. For this purpose pregnant Texel ewes were inoculated intra-amniotically with *Ureaplasma parvum* and serial collections of exhaled breath were performed for 6 days. *Ureaplasma parvum* infection induced a distinct VOC-signature in expired breath of pregnant sheep that was significantly different between day 0 and 1 vs. day 5 and 6. Based on a profile of only 15 discriminatory volatiles, animals could correctly be classified as either infected (day 5 and 6) or not (day 0 and 1) with a sensitivity of 83% and a specificity of 71% and an area under the curve of 0.93. Chemical identification of these distinct VOCs revealed the presence of a lipid peroxidation marker nonanal and various hydrocarbons including n-undecane and n-dodecane. These data indicate that intra-amniotic infections can be detected by VOC analyses of exhaled breath and might provide insight into temporal dynamics of intra-amniotic infection and its underlying pathways. In particular, several of these volatiles are associated with enhanced oxidative stress and undecane and dodecane have been reported as predictive biomarker of spontaneous preterm birth in humans. Applying VOC analysis for the early detection of intra-amniotic infections will lead to appropriate surveillance of these high-risk pregnancies, thereby facilitating appropriate clinical course of action including early treatment of preventative measures for pre-maturity-associated morbidities.

## Introduction

Chorioamnionitis, inflammation associated with an intra-uterine infection of the amniotic fluid and fetal membranes is a major risk factor for preterm birth and an independent risk factor for postnatal disorders such as chronic lung disease, necrotizing enterocolitis, and periventricular leukomalacia (1–3). Incidences of chorioamnionitis are inversely related to gestational age (GA) at birth ranging from an incidence of >70% at 24 weeks GA to 16% at 34 weeks GA (4).

Only a small proportion of pregnant women with preterm birth show clinical signs of chorioamnionitis such as maternal fever, uterine fundal tenderness, maternal tachycardia, fetal tachycardia, and purulent or foul amniotic fluid (5). However, preterm birth is most frequently the result of a clinically unapparent histological chorioamnionitis (6, 7). In these cases, evidence for the presence of chorioamnionitis becomes available only after delivery. More precisely, post-partum histological examination of the placenta with evidence of inflammation and necrosis throughout the chorionic plate and amnion is currently the gold standard to diagnose chorioamnionitis (5).

There is emerging evidence that the timing and duration of intra-amniotic infections are crucial determinants for developmental injury at birth (8, 9). Moreover, we and others have successfully tested *in utero* therapies to prevent or treat chorioamnionitis-induced lung and gut injury (10–12). These combined findings highlight that identification of chorioamnionitis at the earliest time point, being *in utero* is essential for optimal treatment and prevention of organ injury during pregnancy or after birth. Collectively, early identification of chorioamnionitis during pregnancy will extend the time window for clinicians to decide and institute the appropriate clinical course of action to (1) prevent of delay preterm birth and (2) improve outcomes for preterm born neonates.

In recent years, there is increasing attention for non-invasive biomarkers. Exhaled breath consists of volatile organic compounds (VOCs), which are formed during various inflammatory and metabolic processes on a cellular and systemic level (13). In adults and older children, the study of metabolomics using exhaled VOCs is known to be such a safe and non-invasive procedure to evaluate ongoing processes of inflammation and oxidative stress (14), which are key requirements for the induction of cystic fibrosis, asthma and chronic obstructive pulmonary disease (15–17). In addition, profiling exhaled VOCs has been applied successfully in the diagnosis of chronic diseases of the intestine (18, 19), liver (20), kidney (21), pancreas (diabetes) (22, 23) and neurodegenerative diseases (24–26).

Using a well-established pre-clinical model of chorioamnionitis, we questioned whether an intra-amniotic infection could be accurately diagnosed by a specific VOC profile in exhaled breath of pregnant sheep.

## Methods and Materials

### Animals

The animal study, including sampling of exhaled breath, and experimental protocols were in line with the guidelines for animal experiments and approved by the Central Authority for Scientific Procedures on Animals and the animal welfare body of Maastricht University. For this proof-of-concept study, we did not perform a power calculation. Within the context of good laboratory animal practice and the 3R principle we did not plan a separate animal study but rather made use of sheep that destined for another study published by Hütten et al. (27). Power calculations were performed for this initial study and a power of *n* = 6 per experimental group was considered adequate.

Six date-mated Texel ewes (*n* = 5 singleton; *n* = 1 twin pregnancy) underwent ultrasound-guided intra-amniotic injection with 5.0^*^10^5^ color-changing units (CCU) of *Ureaplasma parvum* (UP) (strain HPA5) as reported previously (12, 27). In case of twin pregnancy, both uterine horns were inoculated with UP. Amniotic fluid was collected prior to UP injection and during Cesarean section and cultured for UP quantification (28). All animals were group-housed in the same stable under standardized conditions with a 12-h dark/light cycle and had *ad libitum* access to food and water. During sampling procedure, the animals were restrained by experienced care takers to avoid stress. Six days after intra-amniotic injection (129 days GA) fetuses were delivered preterm by Cesarean section under general anesthesia and used for experimental protocols detailed previously (27). At birth, no sex (3 males and 4 females) and bodyweight (3,270 ± 611 g) differences were observed between all lambs.

### Exhaled Breath Sampling

**Figure 1:**
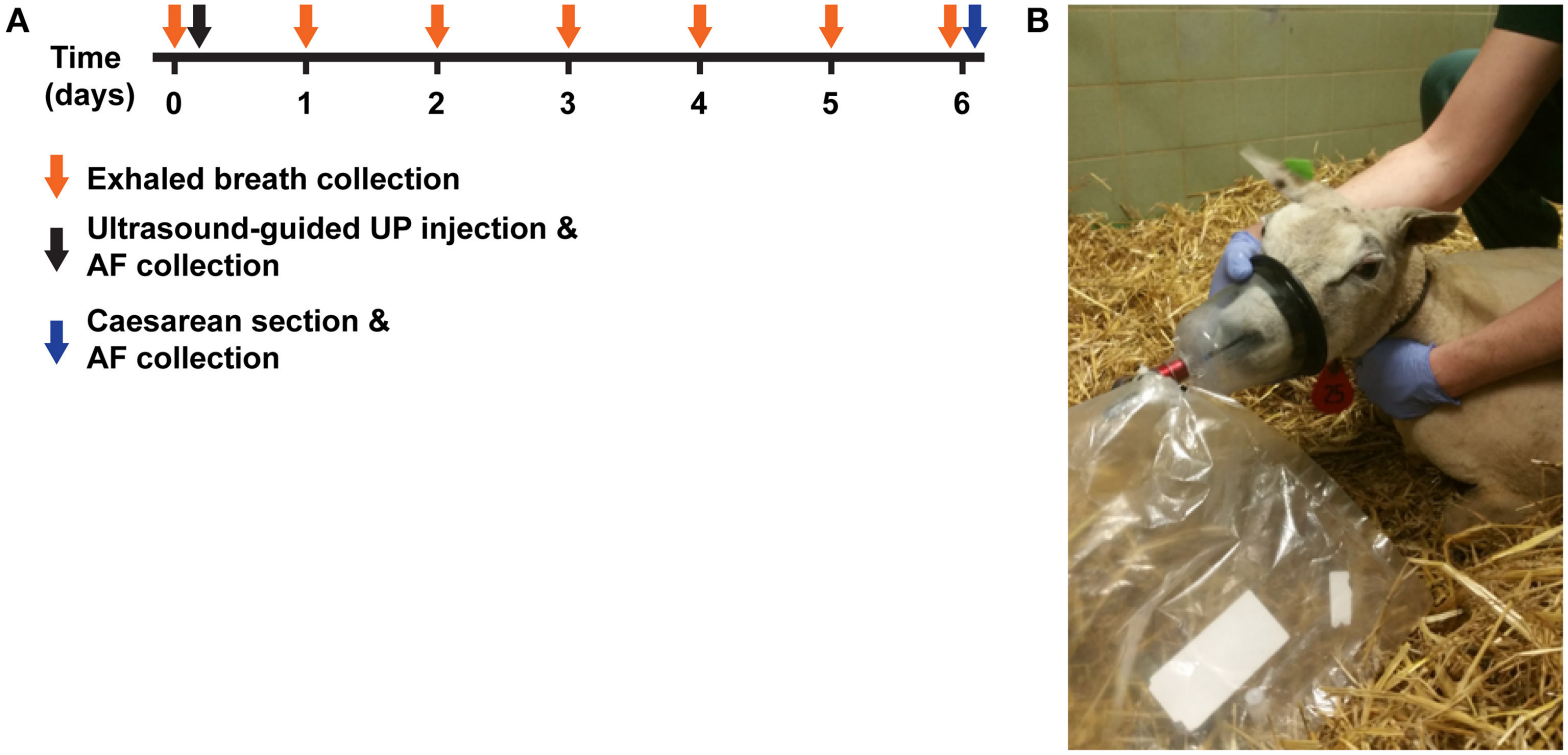
Experimental design. **(A)** Time-mated Texel ewes were given an intra-amniotic injection of UP (5.0*10^5^ CCU). Prior to (*t* = 0) and daily for 6 days after injection, exhaled breath was collected by inflation of 5L Tedlar back through a mouth-nose mask connected to a two-way non-rebreathing valve. Six days (*t* = 6) after intra-amniotic UP-exposure, fetus were delivered by Cesarean section and used for experimented reported elsewhere (27). Group 1 (*n* = 4) was sampled first, followed by group 2 at a later time point. **(B)** Illustrative picture of exhaled breath collection in UP-exposed sheep. VOC, volatile organic compound; UP, *U. Parvum*.

Exhaled breath was collected prior to injection, for baseline (control) measurements, and then daily for 6 days (Figure 1A) after injection. As such, each sheep serves as its own internal reference. During breath collection, animals were monitored for physical signs of eructation. In case of eructation, the sample was discarded as this might negatively influence the VOC profile (29). Breath was collected *via* a customized veterinary mouth-nose mask (Koo Medical Equipment, Arese, Italy) connected to a non-rebreathing valve (Ruben Valve, Intersurgical Ltd., Wokingham, Berkshire UK) and an inert 5L Tedlar (Tedlar Bag, SKC Ltd., Dorset, UK) gas-sampling bag (Figure 1B). Immediately after breath sampling, the contents of the bag were transferred onto a stainless steel two-bed desorption tube filled with carbograph 1TD/Carbopack X (Markes International, Llantrisant Business Park, UK) using a pump at a constant flow of = 200 ml/min. The tubes were then air-tight capped and stored at room temperature until further analysis. Between exhaled breath collections, the sampling bags and masks were flushed three times with high-grade nitrogen to ensure that all contaminants were eliminated.

The samples collected at each time point from different animals were collected at the same location in a random order to minimize confounding variable error mediating bias. Timing of exhaled breath collections was between 09.00 and 11.00 to minimize diurnal variations between samplings.

The current study involved two groups of animals, a discovery set to define the parameters and a separate confirmation set to test the rigor of the selected discriminators. All animals followed the same protocol for modeling experimental chorioamnionitis and exhaled breath sampling.

### Analysis of Exhaled Breath Samples

The volatile metabolites in exhaled breath were measured using gas chromatography time-of-flight mass spectrometry (GC-*tof*-MS). All samples were measured in random order.

Before the measurements by GC-*tof*-MS, all sorbent tubes were purged for 5 min to remove water. To remove the volatile metabolites trapped on the sorption tubes automated thermal desorption TD 100 for industry standard (Markes International, Llantrisant, Wales, UK) under a flow of helium was used. The tubes were heated at 270°C to release the VOCs. The vapor containing the released VOCs was then divided into two parts. The first part, consisting of 25% of the released VOCs, was collected in a cold trap at 5°C, whereas the other 75% was re-collected into the identical stainless steel two-bed desorption tube. Of the part of VOCs sample collected in the cold trap, 75% was injected in the Gas Chromatogram column at a temperature of 300°C and separated by capillary gas chromatography (column: RTX-5ms, 30 m × 0.25 mm 5% diphenyl, 95% dimethylsiloxane, film thickness 1 m; Trace 1300GC, Thermo Fisher Scientific, Waltham, Massachusetts). The temperature gradient for the gas chromatograph was programmed in the following manner: 40°C for 5 min, then raised with 10°C/min to a maximum temperature of 270°C, which was maintained for 5 min. Time-of-flight mass spectrometry (*tof*-MS; Bench TOF-dx, Almsco International, Llantrisant, Wales, UK) was used to detect and identify compounds available in the samples. Electron ionization mode was set at 70 eV and the mass range 35–350 m/z was measured. Sample frequency of the mass spectrometer was set to 5 scans/s and analysis run time to 33 min. Following this procedure, a chromatogram was generated for each breath sample of each animal.

### Data Pre-processing and Statistical Analysis

The raw chromatograms obtained by GC-*tof*-MS were first pre-processed to diminish the effect of various artifacts including noise and baseline, column bleeding, and chromatographic shifts. The detailed description of data pre-processing steps can be found elsewhere (30). The GC-*tof*-MS data after noise removal and baseline correction were further transformed by calculating the area under the peak. These calculated areas for each peak were matched from sample to sample based on the similarity in retention time and Pearson correlation between the mass spectra. A high correlation (>85%) was used to consider peaks as the same compounds. The last step was the creation of a data matrix with samples/animals in rows and relative concentrations of measured volatiles metabolite (ion counts) in columns.

Data obtained for group 1 and group 2 consisted of baseline measurements, pre-injection samples, and six consecutive post-injection sampling days (labeled T1, T2, T3, T4, T5, and T6, Figure 1). The animals from group 1 was used as the discovery set, i.e., finding specific VOCs related to chorioamnionitis, while animals in group 2 served as an independent test set i.e., evaluating the predictive power of the selected exhaled VOCs.

The statistical analysis consisted of performing random forest (RF) analysis (31) with 500 trees using exhaled breath samples of group 1. The classification model comprised of finding the exhaled breath VOCs capable to discriminate pre-infection samples (baseline) and post-infection samples corresponding to development of chorioamnionitis. The classification model was first internally validated using so called out-of-bag (oob) samples. For each RF tree one-third of the training samples were left out and not used in the construction of the classification model. The most discriminatory exhaled VOCs were selected looking at the importance index obtained in RF model (32). The final RF model containing the set of the most discriminatory VOCs was validated using independent test set consisting of samples of group 2. The final performance of the model was expressed in sensitivity, specificity and receiver operating characteristic curve (ROC). To visualize the differences between pre-infection and post-infection samples Principal Component Analysis (PCA) score plot was used. PCA is a workhorse technique within various -omics related fields. This technique reduces data dimensionality by means of Principal Components (PCs), while preserving as much variability as possible. The PCs are the linear combination of the original parameters (here VOCs measured in exhaled breath). The PCA score plot enables representing possible trends, groupings and outliers in the data.

## Results

### Intra-Amniotic Infection

**Figure 2:**
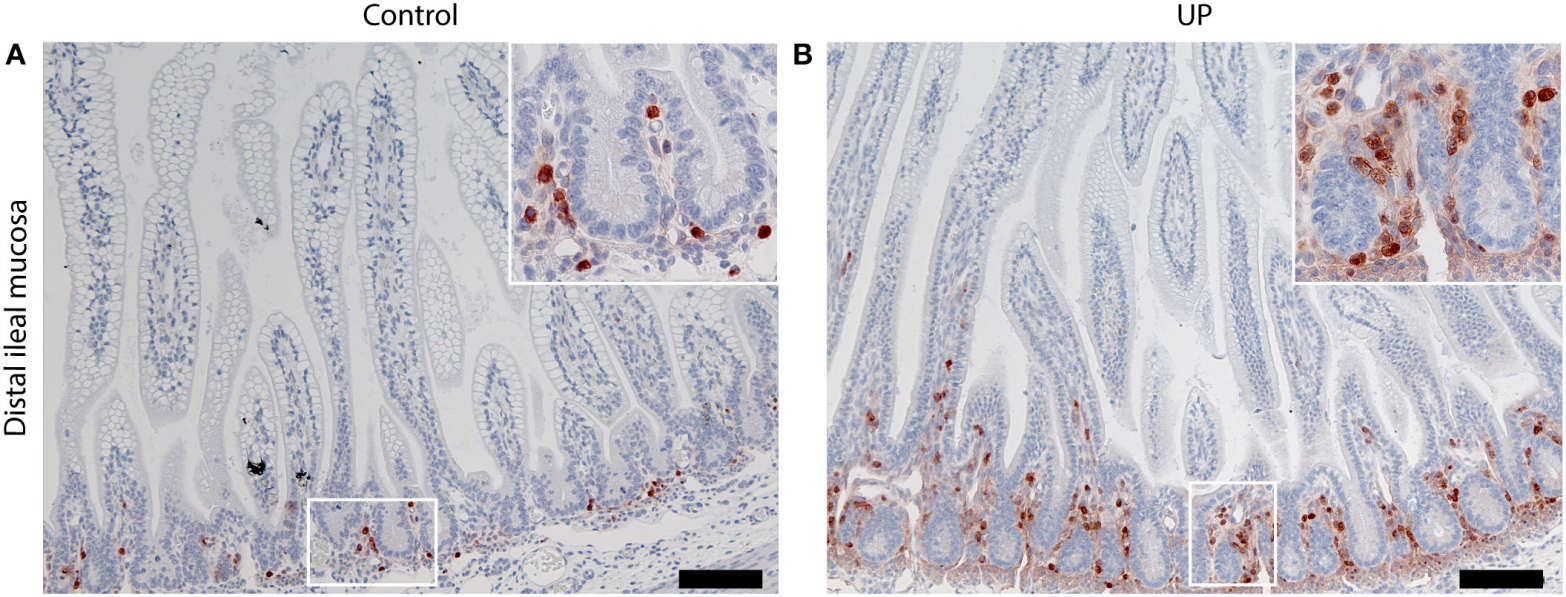
Intra-amniotic UP-exposure leads to gut inflammation. **(A)** In control fetuses, MPO-positive cells were predominantly located in the lower crypt region. **(B)** Six days after intra-amniotic UP-exposure, increased numbers MPO positive cells were observed in the lower crypt. In addition, influx of MPO positive cells into the villi was observed following UP-exposure, which was absent in non-infected historical controls. Images were taken at 100 times magnification, scale bar represents 200 μm. Insert images were taken at 400 times magnification. MPO, myeloperoxidase; UP, *U. Parvum*.

In accordance with several earlier studies (12, 33–35), intra-amniotic infection following intra-amniotic UP administration was confirmed by the presence of UP in amniotic fluid during Cesarean section. No endogenous UP was detected in the amniotic fluid prior to inoculation (data not shown). Experimental induction of chorioamnionitis following intrauterine inoculation of UP is a well-established model and has been shown to induce systemic organ inflammation, including in the intestine (12, 36). Consistent with these earlier reports, gut inflammation at 6 days post UP infection was detected, confirming the phenotype of the model (Figure 2).

### Exhaled Breath

In this study 544 different VOCs were detected in exhaled breath of 4 animals. To increase the group size, the possibility of combining the baseline and T1 samples to define pre-infection class and T5 and T6 time points into post-infection class was investigated. The differences in breath profile between baseline and T1 samples as well as between T5 and T6 samples were investigated by regularized multivariate analysis of variance (rMANOVA). The rMANOVA indicated no statistical differences between baseline and T1 samples (*p*-value of 0.15) as well as between T5 and T6 samples (*p*-value of 0.12).

**Figure 3:**
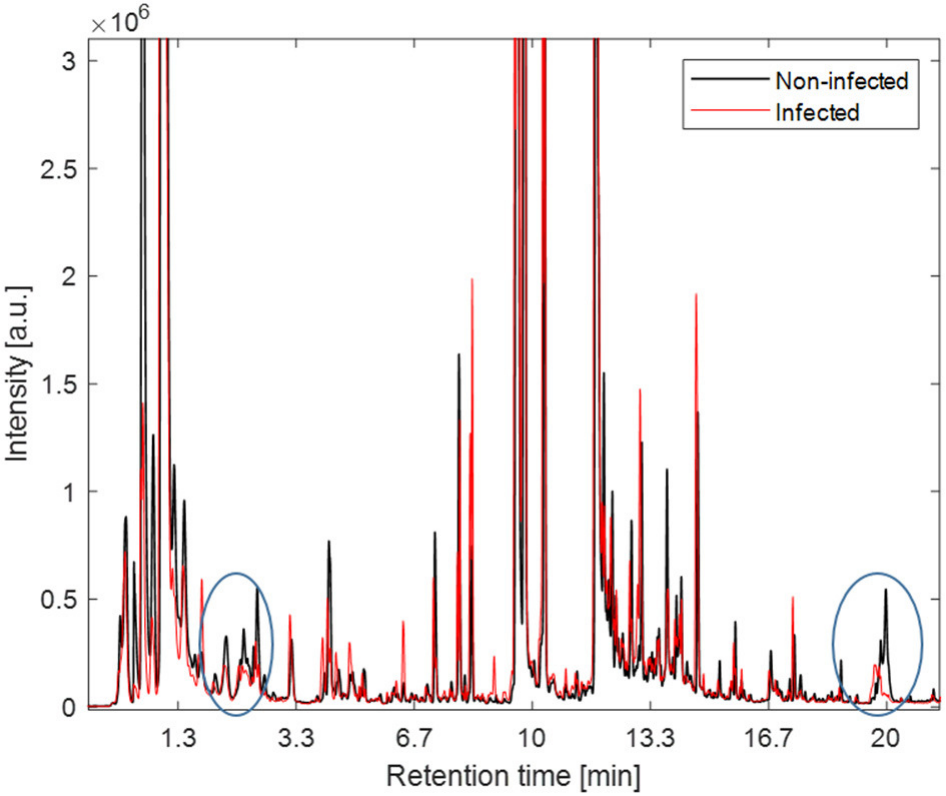
The averaged VOCs profiles for pre-infection (black) and post-infection samples (red). The largest differences in averaged profiles is seen at retention time (RT, i.e., time after compounds captured in exhaled breath pass through a chromatographic column) 2 min and 20 indicated by circles.

In Figure 3 the averaged VOC profiles for pre-infection (baseline samples) and post-infection (T6) samples are shown. As could be expected the VOC profiles of baseline samples, i.e., exhaled breath taken before intra-amniotic UP injection and samples taken at T6, i.e., 6 days after intra-amniotic UP injection were similar. Note, that here the overall averaged profile is visually assessed. The largest differences can be seen in the chromatograms at retention time two and 20 min.

**Figure 4:**
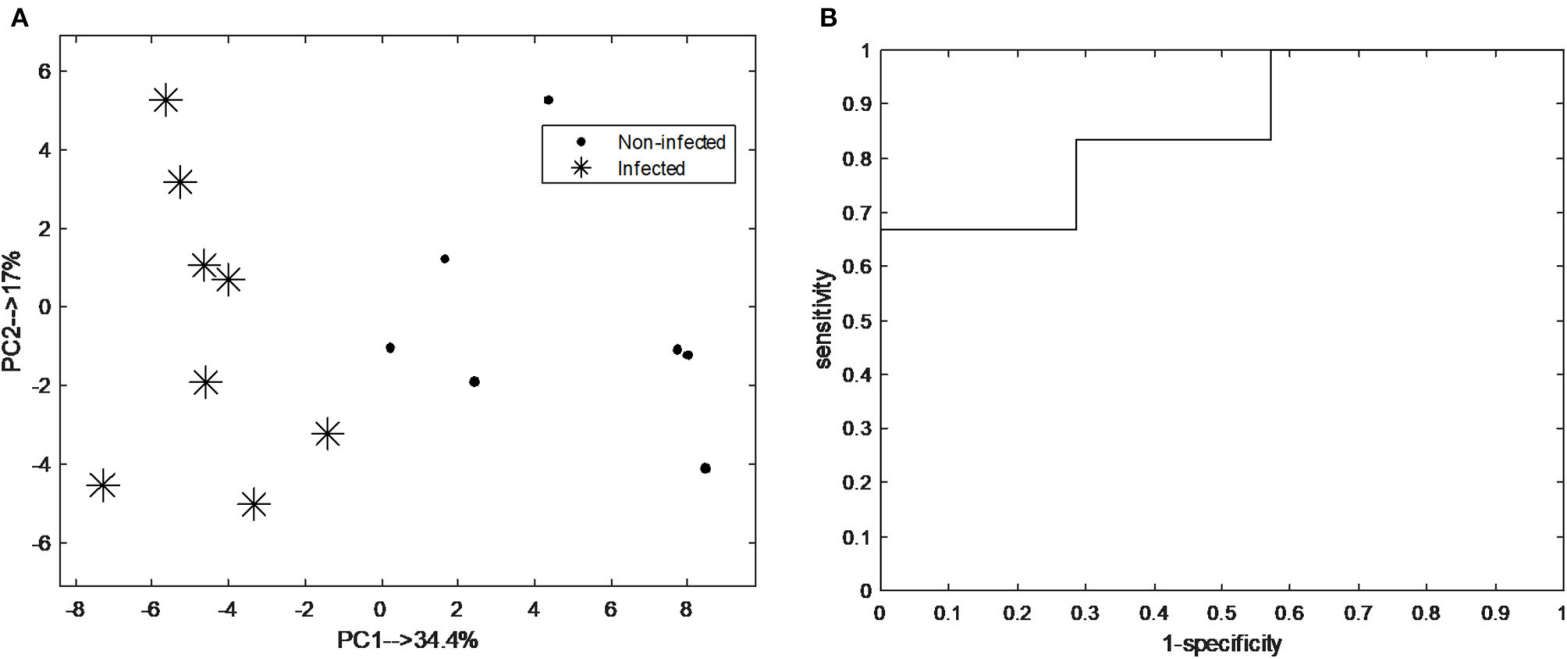
**(A)** Principal component analysis (PCA) score plot score plot obtained from breathogram belonging to pre-infection (i.e., baseline; asterisk) and post-infection (T5-6; black dot) using a set of 15 discriminatory VOCs; **(B)** Receiver operating characteristic curve (ROC) of the independent test samples of the final random forest (RF) model obtained pre-infection and post-infection samples using the set of 15 discriminatory VOCs. The area under the curve is 0.93.

**Figure 5:**
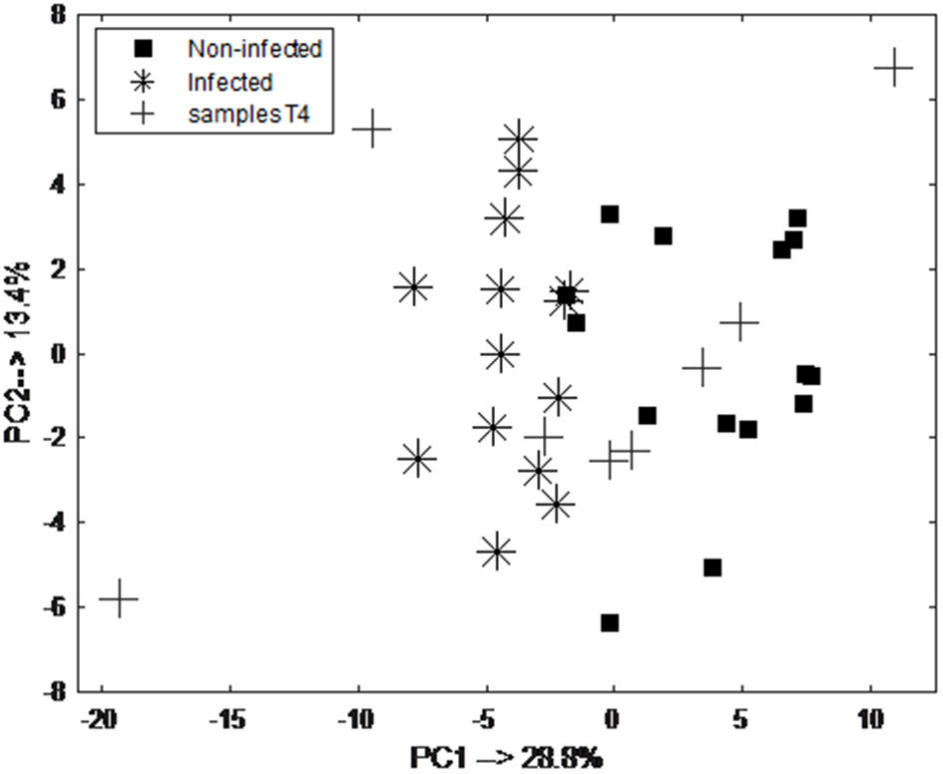
PCA score plot obtained from breathogram belonging to pre-infection (day 0 and 1; black square) and post-infection (day 5 and 6; asterisk) samples and projected samples of animals at day 4 (T4; cross) using a set of 15 discriminatory VOCs. As can be seen majority of the samples are projected in the space belonging to the post-infection samples.

The classification model let to the selection of 15 exhaled VOCs. The final RF model was built using the most discriminatory set of 15 VOCs. In order to visualize the differences between pre-infection (baseline and T1) and post-infection (T5 and T6) samples, PCA analysis was performed using the set of 15 discriminatory VOCs. The corresponding PCA score plot is shown in Figure 4A. As can be seen the pre-infection and post-infection samples are separated among PC1, which explains almost 35% of the data variance (i.e., information contained in the data). Since PC1 always explains the majority of the variance in the data, this result indicates that it corresponds to the differences in the development of infection. The validity of the selected set of 15 discriminatory compounds was tested using the independent test set, consisting of new group of animals sampled following the same protocol as the discovery group (group 2). The external validation of the findings resulted in the ROC curve with an area of 0.93 and sensitivity and specificity of 83.3 and 71.4%, respectively (Figure 4B). The previous time points, T2 and T3, did not show a clear distinction of UP infection using the set of 15 VOCs. The following time point, T4, showed good accuracy of predicting UP infection with accuracy of 0.7. The PCA score plot obtained for pre-infection and post-infection data with projected samples from T4 is shown in Figure 5.

The putative chemical identification of the set of the 15 most discriminatory VOCs was performed using spectrum recognition using the National Institute of Standards and Technology library in combination with spectrum interpretation by an experienced mass-spectrometrist and identification based on retention times of compounds. From a set of 15 VOCs it was possible to chemically identify 11 of them while four remain unknown. These 4 compounds could not be identified due to insufficient mass spectrum, overlap in the retention time or absence of mass spectrum in the library. Moreover, six compounds could be only identified as their global formula and the exact chemical structure remains uncharacterized. Table 1 shows the list of 15 identified VOCs and their relative concentrations change in breath samples obtained from pre-infection and post-infection samples. Up or down regulation of relative VOC concentration is indicated as (+) or (–), respectively, with reference to the infected animal. As can be observed the discriminatory compounds belong mostly to different alkanes and aldehydes.

**Table 1:** A list of 15 discriminatory VOCs, their putative identification, statistical significance (indicated as p-value obtained from rank test after correction for multitesting with Benjamin-Hochberg) and their relative concentration change in post-infection group with respect to pre-infection samples.

**Nr**.	**Compound name**	**Compound change in infected group**	***p*-value**
1	1-butanol	(–)	0.22
2	Nonanal^*^	(+)	0.02
3	n-undecan^*^	(+)	0.02
4	n-dodecan	(+)	0.06
5	6-tridecene	(+)	0.09
6	C_10_H_18_O	(+)	0.07
7	C_10_H_14_	(+)	0.07
8	C_11_H_24_ (branched)^*^	(–)	0.02
9	C_15_H_24_	(+)	0.98
10	C_9_H_18_ (branched)	(+)	0.07
11	C_5_H_7_COOH	(+)	0.15
12	Unknown	(–)	0.98
13	Unknown^*^	(+)	0.04
14	Unknown^*^	(+)	0.02
15	Unknown^*^	(–)	0.03

* *Corresponds to compounds statistically significant*.

To further investigate whether the relative amounts of the individual VOCs are significantly different between pre-infection and post-infection samples, a rank test was used. The corresponding *p*-values are indicated in Table 1. As can be observed the discriminatory compounds belong mostly to different alkanes, alcohol and aldehydes, with the relative concentration of the majority of the compounds being elevated in exhaled breath of the post-infection samples.

### Discussion/Conclusion

Chorioamnionitis is an important risk factor for preterm birth and prematurity-associated disorders such as bronchopulmonary dysplasia, necrotizing enterocolitis, and neurological injury (1, 3, 37). Currently, chorioamnionitis is diagnosed by postnatal histological assessment of the placenta and membranes. Consequently, data on the presence of chorioamnionitis become available postnatally, and do not provide information on timing of onset and duration of infection. This latter aspect is of key importance since information on timing and duration of infection are critical determinants for initiation of optimal treatment regimes in preterm infants. Moreover, timely diagnosis and treatment of intra-amniotic infections as the main underlying mechanisms of preterm birth using antibiotic treatment and fetal delivery has shown to improve maternal and neonatal outcomes (38).

Several pre-clinical studies showed that interventions at the earliest possible moment, being *in utero*, have been successfully tested (10–12, 39–44). Successful development and implementation of such treatment strategies in clinical care is currently impeded by a lack of diagnostic tools for early (antenatal) detection and surveillance of intra-amniotic infections. As such, we aimed to develop a diagnostic tool for early (antenatal) recognition of this distinct risk factor *in utero* in a safe and non-invasive manner.

In the current proof-of-concept study, we demonstrated that intra-amniotic UP infection induces a distinct VOC-signature in exhaled breath of pregnant sheep. Based on the identified profile of 15 discriminatory volatiles, animals could correctly be classified as either infected or not with good sensitivity (83.3%) and specificity (71.4%). Importantly, we were capable of differentiating UP infection with very good accuracy of 0.7 from 4 post-infection onwards. The prediction performance of UP infection by the VOCs profile was indeterminate 72 h post-infection (data not shown). Those findings indicate that the UP infection can be detectable with good accuracy using exhaled breath profile from 96 h onwards post-infection. The discovery of a distinct VOC profile to determine and monitor an intra-amniotic UP infection extends recent clinical studies by Lacey et al. in which they demonstrated that VOC testing of vaginal swabs can be used to predict group B streptococcus infection during pregnancy (45) and detect bacterial vaginosis which was linked to preterm labor (46).

Among the identified VOCs associated with UP-infection are various alkenes, aldehyde and alcohols which are primary and secondary products of the lipid peroxidation. The elevated level of the lipid peroxidation marker nonanal and various hydrocarbons including the identified n-undecane and n-dodecane is in line with the concept that intra-uterine infection induces higher level of oxidative stress. The presence of these volatiles indicates the occurrence of oxidative stress, a process defined as an imbalance between the production of and the protection against reactive oxygen species in favor of the first (47) and associated with inflammation (48). Chorioamnionitis-induced oxidative stress has been shown to play an important role in preterm labor (49) and its occurrence has recently been underlined in a human study associating placental lesions due to chorioamnionitis with higher levels of oxidative stress biomarkers in cord blood of neonates (50). More closely to our setup, the induction of systemic oxidative stress and modest lung oxidative stress, potentially contributing to bronchopulmonary dysplasia (BPD), by intra-amniotic endotoxin has been demonstrated in fetal lambs (51, 52). This alleged involvement of oxidative stress in chorioamnionitis-induced preterm labor and potential development of BPD implies prospective antioxidant supplementation might be a good strategy in the future to protect preterm newborns from severe lung damage (48). This approach highlights the role of antenatal identification of women at risk of chorioamnionitis using the non-invasive markers, such as VOC profiles in breath.

Interestingly, due to the direct link between inflammation and oxidative stress, the volatile undecane has been associated with both processes in general. This is especially the case for infections caused by viruses though, as they do not produce their own metabolites but instead hijack the metabolism of the host, thereby enhancing processes such as glycolysis and oxidative stress (53). Although various studies have already been performed to identify volatile markers unique for specific bacterial strains, n-undecane has not yet been reported as possible biomarker specific for any of the pathogens tested until now (54–56). However, UP has not been included in such investigations thus far, indicating it might be useful to investigate whether undecane is indeed specifically produced by this pathogen or a byproduct of the oxidative stress induced by this bacterium instead.

Finally, the volatiles undecane and dodecane that were discriminative between pre-infection and post-infection samples in our study have recently been reported as predictive biomarker of spontaneous preterm birth in humans (57). In that study, the maternal serum metabolome was studied at 15- and 20-weeks' gestation in an attempt to identify biomarkers for an increased risk of spontaneous preterm birth, which may lead to enhanced neonatal morbidities including BPD, in asymptomatic nulliparous women in 2 different cohorts. In one of the cohorts, the alkanes undecane, dodecane, and decane were associated with spontaneous preterm birth whereas these elevated levels could not be observed in the other cohort. Consequently, the study did not provide enough evidence yet to use these alkanes as clinical predictor but does underline once more the relationship between oxidative stress and the risk of neonatal morbidity and mortality (57).

Strengths of the current study are (1) the controlled environment of the established model that is free of confounding factors including behavioral and dietary influences; (2) establishment of individual baseline measurements prior to intra-amniotic infection; (3) the use of a precisely regulated amount of UP, which are the most common microorganisms isolated in cases of spontaneous (asymptomatic) preterm birth (58–60) and significantly associated with histological chorioamnionitis (60); (4) the use of a sampling technique that is used in clinical practice; and (5) the use of a simple, non-invasive, and repeatedly obtained sample medium, being exhaled air, which will appeal to patients. A limitation is that a number of VOCs remained incompletely characterized. Still, the identified VOCs could serve as specific markers for chorioamnionitis, and UP infections specifically, and could thus be applied for future human exhaled breath studies. Future pre-clinical studies should extend on the current findings and look for further discriminative markers by (1) increasing the experimental power; (2) taking the polymicrobial nature of intra-amniotic infections into account; and (3) focus on discerning timing of onset of infection or duration of infection, which are essential elements that determine organ outcomes at birth. Moreover, combining exhaled VOCs with peri-partum diagnostic tools, including amniotic fluid parameters, might aid in the diagnosis of (subclinical) chorioamnionitis. Furthermore, extending the time window of VOC profiling post-partum might provide essential insight into maternal recovery from chorioamnionitis and contribute to risk profiling for women at risk of developing post-partum endometritis, which is the most common post-partum complication following chorioamnionitis, thereby allowing selective use of prophylactic antibiotics (61, 62).

## Conclusion

With 15 million cases globally, preterm birth remains a major health care problem (63) with chorioamnionitis as its most important risk factor. Analyses of VOCs in exhaled breath of pregnant sheep shows great potential to identify pregnancies complicated by intra-amniotic infections and clinical implementation would be an immense breakthrough in perinatal diagnostics. Early detection of intra-amniotic infections with point-of-care VOC testing, potentially combined with peri-partum amniotic fluid biomarkers indicative for intra-amniotic infection (64), will lead to optimal surveillance of these high-risk pregnancies and will facilitate appropriate clinical management including antibiotic treatment and timely treatment or preventative measures for pre-maturity-associated morbidities. A clinical study in a high risk pregnancy population, defined by (1) chorioamnionitis in previous pregnancies; (2) asymptomatic cervical shortening; and (3) prolonged rupture of membranes has been planned to verify the results from this proof-of-concept study in combination with intra- and post-partum diagnostic techniques that are currently in practice, including clinical parameters, amniotic fluid parameters, and placenta histology (5, 64).

## Data Availability Statement

The raw data supporting the conclusions of this article will be made available by the authors, without undue reservation.

## Ethics Statement

The animal study was reviewed and approved by Central Authority for Scientific Procedures on Animals and the animal welfare body of Maastricht University.

## Author Contributions

DO, AB, and TW conceived original idea. DO and MH performed ultrasound-guided injections with UP, which was provided by OS. DO collected and processed exhaled breath for laboratory analyses. AB, AS, and F-JS supervised laboratory analyses on exhaled breath. DO, AB, MH, SA-N, RJ, OS, F-JS, AS, and TW contributed to the interpretation of the results. DO and AB wrote the manuscript with input from all authors. DO and TW supervised the project. All authors read, and approved the submitted version.

## Conflict of Interest

The authors declare that the research was conducted in the absence of any commercial or financial relationships that could be construed as a potential conflict of interest.

## References

[1] Jobe AH Bancalari EJ. Bronchopulmonary dysplasia. Am J Respir Crit Care Med. (2001) 163:1723–9. 10.1164/ajrccm.163.7.2011060 11401896

[2] Volpe JJ. The encephalopathy of prematurity–brain injury and impaired brain development inextricably intertwined. Semin Pediatr Neurol. (2009) 16:167–78. 10.1016/j.spen.2009.09.005 19945651 PMC2799246

[3] Been JV Lievense S Zimmermann LJ Kramer BW Wolfs TG. Chorioamnionitis as a risk factor for necrotizing enterocolitis: a systematic review and meta-analysis. J Pediatr. (2013) 162:236–42.e232. 10.1016/j.jpeds.2012.07.012 22920508

[4] Lahra MM Jeffery HEJ. A fetal response to chorioamnionitis is associated with early survival after preterm birth. Am J Obstet Gynecol. (2004) 190:147–51. 10.1016/j.ajog.2003.07.012 14749651

[5] Tita AT Andrews WW. Diagnosis and management of clinical chorioamnionitis. Clin Perinatol. (2010) 37:339–54. 10.1016/j.clp.2010.02.003 20569811 PMC3008318

[6] Hagberg H Wennerholm U-B Sävman K. Sequelae of chorioamnionitis. Curr Opin Infect Dis. (2002) 15:301–6. 10.1097/00001432-200206000-00014 12015466

[7] Romero R Gotsch F Pineles B Kusanovic JP. Inflammation in pregnancy: its roles in reproductive physiology, obstetrical complications, fetal injury. Nutr Rev. (2007) 65:S194–202. 10.1111/j.1753-4887.2007.tb00362.x 18240548

[8] Gussenhoven R Westerlaken RJ Ophelders DR Jobe AH Kemp MW Kallapur SG. Chorioamnionitis, neuroinflammation, and injury: timing is key in the preterm ovine fetus. J Neuroinflammation. (2018) 15:113. 10.1186/s12974-018-1149-x 29673373 PMC5907370

[9] Heymans C De Lange I Lenaerts K Kessels L Hadfoune M Rademakers G. Chorioamnionitis induces enteric nervous system injury: effects of timing and inflammation in the ovine fetus. Mol Med. (2020) 26:1–10. 10.1186/s10020-020-00206-x 32883198 PMC7469100

[10] Nikiforou M Vanderlocht J Chougnet CA Jellema RK Ophelders DR Joosten M. Prophylactic interleukin-2 treatment prevents fetal gut inflammation and injury in an ovine model of chorioamnionitis. Inflamm Bowel Dis. (2015) 21:2026–38. 10.1097/MIB.0000000000000455 26002542

[11] Willems MG Ophelders DR Nikiforou M Jellema RK Butz A Delhaas T. Systemic interleukin-2 administration improves lung function and modulates chorioamnionitis-induced pulmonary inflammation in the ovine fetus. Am J Physiol Lung Cell Mol Physiol. (2015) 310:L1–7. 10.1152/ajplung.00289.2015 26519206

[12] Van Gorp C De Lange IH Spiller OB Dewez F Cillero Pastor B Heeren R. Protection of the ovine fetal gut against ureaplasma-induced chorioamnionitis: a potential role for plant sterols. Nutrients. (2019) 11:968. 10.3390/nu11050968 31035616 PMC6566982

[13] Boots AW Bos LD Van Der Schee MP Van Schooten F-J Sterk PJ. Exhaled molecular fingerprinting in diagnosis and monitoring: validating volatile promises. Trends Mol Med. (2015) 21:633–44. 10.1016/j.molmed.2015.08.001 26432020

[14] Mazzatenta A Pokorski M Di Giulio C. Real time analysis of volatile organic compounds (VOCs) in centenarians. Respir Physiol Neurobiol. (2015) 209:47–51. 10.1016/j.resp.2014.12.014 25542135

[15] Mazzatenta A Di Giulio C Pokorski M. Pathologies currently identified by exhaled biomarkers. Respir Physiol Neurobiol. (2013) 187:128–34. 10.1016/j.resp.2013.02.016 23485801

[16] Van De Kant KD Van Berkel JJ Jöbsis Q Passos VL Klaassen EM Van Der Sande L. Exhaled breath profiling in diagnosing wheezy preschool children. Eur Respir J. (2013) 41:183–8. 10.1183/09031936.00122411 23277518

[17] Smolinska A Klaassen EM Dallinga JW Van De Kant KD Jobsis Q Moonen EJ. Profiling of volatile organic compounds in exhaled breath as a strategy to find early predictive signatures of asthma in children. PLoS ONE. (2014) 9:e95668. 10.1371/journal.pone.0095668 24752575 PMC3994075

[18] Bodelier AG Smolinska A Baranska A Dallinga JW Mujagic Z Vanhees K. Volatile organic compounds in exhaled air as novel marker for disease activity in Crohn's disease: a metabolomic approach. Inflamm Bowel Dis. (2015) 21:1776–85. 10.1097/MIB.0000000000000436 26199990

[19] Arasaradnam RP Mcfarlane M Daulton E Skinner J O'connell N Wurie S. Non-invasive exhaled volatile organic biomarker analysis to detect inflammatory bowel disease (IBD). Dig Liver Dis. (2016) 48:148–53. 10.1016/j.dld.2015.10.013 26682719

[20] Pijls KE Smolinska A Jonkers DM Dallinga JW Masclee AA Koek GH. A profile of volatile organic compounds in exhaled air as a potential non-invasive biomarker for liver cirrhosis. Sci Rep. (2016) 6:19903. 10.1038/srep19903 26822454 PMC4731784

[21] Obermeier J Trefz P Happ J Schubert JK Staude H Fischer D-C. Exhaled volatile substances mirror clinical conditions in pediatric chronic kidney disease. PLoS ONE. (2017) 12:e0178745. 10.1371/journal.pone.0178745 28570715 PMC5453591

[22] Minh TDC Blake DR Galassetti PR. The clinical potential of exhaled breath analysis for diabetes mellitus. Diabetes Res Clin Pract. (2012) 97:195–205. 10.1016/j.diabres.2012.02.006 22410396 PMC3384765

[23] Mazzatenta A Pokorski M Di Giulio C. Real-time breath analysis in type 2 diabetes patients during cognitive effort. Adv Exp Med Biol. (2013) 788:247–53. 23835985 10.1007/978-94-007-6627-3_35

[24] Mazzatenta A Pokorski M Sartucci F Domenici L Di Giulio C. Volatile organic compounds (VOCs) fingerprint of Alzheimer's disease. Respir Physiol Neurobiol. (2015) 209:81–4. 10.1016/j.resp.2014.10.001 25308706

[25] Invitto S Mazzatenta A. Olfactory event-related potentials and exhaled organic volatile compounds: the slow link between olfactory perception and breath metabolic response. A pilot study on phenylethyl alcohol and vaseline oil. Brain Sci. (2019) 9:84. 10.3390/brainsci9040084 30991670 PMC6523942

[26] Tiele A Wicaksono A Daulton E Ifeachor E Eyre V Clarke S. Breath-based non-invasive diagnosis of Alzheimer's disease: a pilot study. J Breath Res. (2020) 14:026003. 10.1088/1752-7163/ab6016 31816609

[27] Hütten MC Fehrholz M Konrad FM Ophelders D Kleintjes C Ottensmeier B. Detrimental effects of an inhaled phosphodiesterase-4 inhibitor on lung inflammation in ventilated preterm lambs exposed to chorioamnionitis are dose dependent. J Aerosol Med Pulmonary Drug Delivery. (2019) 32:395–404. 10.1089/jamp.2019.1528 31573405

[28] Miura Y Payne MS Keelan JA Noe A Carter S Watts R. Maternal intravenous treatment with either azithromycin or solithromycin clears *Ureaplasma parvum* from the amniotic fluid in an ovine model of intrauterine infection. Antimicrob Agents Chemother. (2014) 58:5413–20. 10.1128/AAC.03187-14 24982089 PMC4135857

[29] Oertel P Küntzel A Reinhold P Köhler H Schubert JK Kolb J. Continuous real-time breath analysis in ruminants: effect of eructation on exhaled VOC profiles. J Breath Res. (2018) 12:036014. 10.1088/1752-7163/aabdaf 29648550

[30] Smolinska A Hauschild AC Fijten RR Dallinga JW Baumbach J Van Schooten FJ. Current breathomics–a review on data pre-processing techniques and machine learning in metabolomics breath analysis. J Breath Res. (2014) 8:027105. 10.1088/1752-7155/8/2/027105 24713999

[31] Breiman L. Random forests. Machine Learning. (2001) 45:5–32. 10.1023/A:1010933404324

[32] Stavropoulos G Van Vorstenbosch R Van Schooten F Smolinska A. Random Forest and Ensemble Methods. Amsterdam: Elsevier (2020).

[33] Robinson JW Dando SJ Nitsos I Newnham J Polglase GR Kallapur SG. *Ureaplasma parvum* serovar 3 multiple banded antigen size variation after chronic intra-amniotic infection/colonization. PLoS ONE. (2013) 8:e62746. 10.1371/journal.pone.0062746 23638142 PMC3637154

[34] Gussenhoven R Ophelders DRMG Kemp MW Payne MS Spiller OB Beeton ML. The paradoxical effects of chronic intra-amniotic *Ureaplasma parvum* exposure on ovine fetal brain development. Dev Neurosci. (2017) 39:472–86. 10.1159/000479021 28848098 PMC5828963

[35] Kemp MW Ahmed S Beeton ML Payne MS Saito M Miura Y. Foetal *Ureaplasma parvum* bacteraemia as a function of gestation-dependent complement insufficiency: evidence from a sheep model of pregnancy. Am J Reprod Immunol. (2017) 77:e12599. 10.1111/aji.12599 27862576

[36] Wolfs TG Kallapur SG Knox CL Thuijls G Nitsos I Polglase GR. Antenatal ureaplasma infection impairs development of the fetal ovine gut in an IL-1-dependent manner. Mucosal Immunol. (2013) 6:547–56. 10.1038/mi.2012.97 23149664

[37] Volpe JJ. Brain injury in premature infants: a complex amalgam of destructive and developmental disturbances. Lancet Neurol. (2009) 8:110–24. 10.1016/S1474-4422(08)70294-1 19081519 PMC2707149

[38] Johnson CT Adami RR Farzin A. Antibiotic therapy for chorioamnionitis to reduce the global burden of associated disease. Front Pharmacol. (2017) 8:97. 10.3389/fphar.2017.00097 28352229 PMC5348523

[39] Ng PY Ireland DJ Keelan JA. Drugs to block cytokine signaling for the prevention and treatment of inflammation induced preterm birth. Front Immunol. (2015) 6:166. 10.3389/fimmu.2015.00166 25941525 PMC4403506

[40] Keelan JA Payne MS Kemp MW Ireland DJ Newnham JP. A new, potent, and placenta-permeable macrolide antibiotic, solithromycin, for the prevention and treatment of bacterial infections in pregnancy. Front Immunol. (2016) 7:111. 10.3389/fimmu.2016.00111 27066004 PMC4817400

[41] Ophelders DR Gussenhoven R Lammens M Küsters B Kemp MW Newnham JP. Neuroinflammation and structural injury of the fetal ovine brain following intra-amniotic Candida albicans exposure. J Neuroinflamm. (2016) 13:29. 10.1186/s12974-016-0492-z 26842664 PMC4739103

[42] Paton MC Allison BJ Li J Fahey MC Sutherland AE Nitsos I. Human umbilical cord blood therapy protects cerebral white matter from systemic LPS exposure in preterm fetal sheep. Dev Neurosci. (2018) 40:258–70. 10.1159/000490943 30179864

[43] Gomez-Lopez N Romero R Garcia-Flores V Leng Y Miller D Hassan SS. Inhibition of the NLRP3 inflammasome can prevent sterile intra-amniotic inflammation, preterm labor/birth, and adverse neonatal outcomes. Biol Reprod. (2019) 100:1306–18. 10.1093/biolre/ioy264 30596885 PMC6497524

[44] Galinsky R Dhillon SK Dean JM Davidson JO Lear CA Wassink G. Tumor necrosis factor inhibition attenuates white matter gliosis after systemic inflammation in preterm fetal sheep. J Neuroinflamm. (2020) 17:1–16. 10.1186/s12974-020-01769-6 32293473 PMC7087378

[45] Lacey L Daulton E Wicaksono A Covington JA Quenby S. Detection of group B *Streptococcus* in pregnancy by vaginal volatile organic compound analysis: a prospective exploratory study. Transl Res. (2020) 216:23–9. 10.1016/j.trsl.2019.09.002 31585066

[46] Lacey L Daulton E Wicaksono A Covington JA Quenby S. Volatile organic compound analysis, a new tool in the quest for preterm birth prediction-an observational cohort study. Sci Rep. (2020) 10:1–9. 10.1038/s41598-020-69142-4 32699319 PMC7376243

[47] Sies H. Oxidative stress: a concept in redox biology and medicine. Redox Biol. (2015) 4:180–3. 10.1016/j.redox.2015.01.002 25588755 PMC4309861

[48] Perrone S Tataranno ML Buonocore G. Oxidative stress and bronchopulmonary dysplasia. J Clin Neonatol. (2012) 1:109–14. 10.4103/2249-4847.101683 24027702 PMC3762019

[49] Temma K Shimoya K Zhang Q Kimura T Wasada K Kanzaki T. Effects of 4-hydroxy-2-nonenal, a marker of oxidative stress, on the cyclooxygenase-2 of human placenta in chorioamnionitis. Mol Hum Reprod. (2004) 10:167–71. 10.1093/molehr/gah030 14981143

[50] Perrone S Tataranno ML Negro S Longini M Toti MS Alagna MG. Placental histological examination and the relationship with oxidative stress in preterm infants. Placenta. (2016) 46:72–8. 10.1016/j.placenta.2016.08.084 27697224

[51] Cheah FC Jobe AH Moss TJ Newnham JP Kallapur SG. Oxidative stress in fetal lambs exposed to intra-amniotic endotoxin in a chorioamnionitis model. Pediatr Res. (2008) 63:274–9. 10.1203/PDR.0b013e31815f653b 18091343

[52] Song Y Pinniger GJ Bakker AJ Moss TJ Noble PB Berry CA. Lipopolysaccharide-induced weakness in the preterm diaphragm is associated with mitochondrial electron transport chain dysfunction and oxidative stress. PLoS ONE. (2013) 8:e73457. 10.1371/journal.pone.0073457 24039949 PMC3765262

[53] Gould O Ratcliffe N Krol E De Lacy Costello B. Breath analysis for detection of viral infection, the current position of the field. J Breath Res. (2020) 14:041001. 10.1088/1752-7163/ab9c32 32531777

[54] Bos LD Sterk PJ Schultz MJ. Volatile metabolites of pathogens: a systematic review. PLoS Pathog. (2013) 9:e1003311. 10.1371/journal.ppat.1003311 23675295 PMC3649982

[55] Boots AW Smolinska A Van Berkel JJ Fijten RR Stobberingh EE Boumans ML. Identification of microorganisms based on headspace analysis of volatile organic compounds by gas chromatography-mass spectrometry. J Breath Res. (2014) 8:027106. 10.1088/1752-7155/8/2/027106 24737039

[56] Karami N Karimi A Aliahmadi A Mirzajan F Rezadoost H Ghassempour A. Identification of bacteria using volatile organic compounds. Cell Mol Biol (Noisy-le-grand). (2017) 63:112–21. 10.14715/cmb/2017.63.2.18 28364792

[57] Souza RT Mckenzie EJ Jones B De Seymour JV Thomas MM Zarate E. Trace biomarkers associated with spontaneous preterm birth from the maternal serum metabolome of asymptomatic nulliparous women—parallel case-control studies from the SCOPE cohort. Sci Rep. (2019) 9:13701. 10.1038/s41598-019-50252-7 31548567 PMC6757051

[58] Kim CJ Romero R Chaemsaithong P Chaiyasit N Yoon BH Kim YM. Acute chorioamnionitis and funisitis: definition, pathologic features, clinical significance. Am J Obstet Gynecol. (2015) 213:S29–52. 10.1016/j.ajog.2015.08.040 26428501 PMC4774647

[59] Payne MS Ireland DJ Watts R Nathan EA Furfaro LL Kemp MW. *Ureaplasma parvum* genotype, combined vaginal colonisation with Candida albicans, and spontaneous preterm birth in an Australian cohort of pregnant women. BMC Preg Childbirth. (2016) 16:312. 10.1186/s12884-016-1110-x 27756249 PMC5070304

[60] Sweeney EL Dando SJ Kallapur SG Knox CL. The human Ureaplasma species as causative agents of chorioamnionitis. Clin Microbiol Rev. (2017) 30:349–79. 10.1128/CMR.00091-16 27974410 PMC5217797

[61] Venkatesh K Glover A Vladutiu C Stamilio D. Association of chorioamnionitis and its duration with adverse maternal outcomes by mode of delivery: a cohort study. BJOG Int J Obstet Gynaecol. (2019) 126:719–27. 10.1111/1471-0528.15565 30485648

[62] Fowler JR Simon LV. Chorioamnionitis. StatPearls [Internet]. Treasure Island, FL: StatPearls Publishing (2019). Available online at: https://www.ncbi.nlm.nih.gov/books/NBK532251/

[63] Blencowe H Cousens S Chou D Oestergaard M Say L Moller AB. Born too soon: the global epidemiology of 15 million preterm births. Reprod Health 10 Suppl. (2013) 1:S2. 10.1186/1742-4755-10-S1-S2 24625129 PMC3828585

[64] Oh KJ Lee J Romero R Park HS Hong J-S Yoon BH. A new rapid bedside test to diagnose and monitor intraamniotic inflammation in preterm PROM using transcervically collected fluid. Am J Obstet Gynecol. (2020) 223:423.e421–423.e415. 10.1016/j.ajog.2020.02.037 32114081 PMC9521159

